# Gut Microbiota: A Novel Therapeutic Target for Parkinson’s Disease

**DOI:** 10.3389/fimmu.2022.937555

**Published:** 2022-06-24

**Authors:** Manlian Zhu, Xia Liu, Yiru Ye, Xiumei Yan, Yiwen Cheng, Longyou Zhao, Feng Chen, Zongxin Ling

**Affiliations:** ^1^Department of Geriatrics, Lishui Second People’s Hospital, Lishui, China; ^2^Department of Intensive Care Unit, The First Affiliated Hospital, School of Medicine, Zhejiang University, Hangzhou, China; ^3^Department of Respiratory Medicine, Lishui Central Hospital, Lishui, China; ^4^Department of Laboratory Medicine, Lishui Second People’s Hospital, Lishui, China; ^5^Jinan Microecological Biomedicine Shandong Laboratory, Jinan, China; ^6^Collaborative Innovation Center for Diagnosis and Treatment of Infectious Diseases, State Key Laboratory for Diagnosis and Treatment of Infectious Diseases, National Clinical Research Center for Infectious Diseases, The First Affiliated Hospital, School of Medicine, Zhejiang University, Hangzhou, China

**Keywords:** α-synuclein, diet, gut microbiota, microbiota-targeted therapy, Parkinson’s disease

## Abstract

Parkinson’s disease (PD) is the second most common neurodegenerative disease characterized by motor dysfunction. Growing evidence has demonstrated that gut dysbiosis is involved in the occurrence, development and progression of PD. Numerous clinical trials have identified the characteristics of the changed gut microbiota profiles, and preclinical studies in PD animal models have indicated that gut dysbiosis can influence the progression and onset of PD *via* increasing intestinal permeability, aggravating neuroinflammation, aggregating abnormal levels of α-synuclein fibrils, increasing oxidative stress, and decreasing neurotransmitter production. The gut microbiota can be considered promising diagnostic and therapeutic targets for PD, which can be regulated by probiotics, psychobiotics, prebiotics, synbiotics, postbiotics, fecal microbiota transplantation, diet modifications, and Chinese medicine. This review summarizes the recent studies in PD-associated gut microbiota profiles and functions, the potential roles, and mechanisms of gut microbiota in PD, and gut microbiota-targeted interventions for PD. Deciphering the underlying roles and mechanisms of the PD-associated gut microbiota will help interpret the pathogenesis of PD from new perspectives and elucidate novel therapeutic strategies for PD.

## Introduction

Parkinson’s disease (PD) is a chronic, progressive, and disabling neurodegenerative disorder that affects the middle-aged and elderly population. It has been ranked second neurodegenerative disorders worldwide just after Alzheimer’s disease (AD) (1). Since its description over 200 years ago by James Parkinson, PD has shown the fastest rising prevalence of all neurodegenerative diseases worldwide (2, 3). As reported in the Global Burden of Disease Study 2016, the prevalence and incidence of PD are observed in 1–2% of the population aged >60 years, which have not changed significantly in industrialized countries including the United States and UK, between 1990 and 2016 (2, 3). However, the global burden of PD has more than doubled to over 6 million as a result of increasing numbers of older people (4, 5), with potential contributions from environmental factors and longer disease duration. With the remarkable evolution of China’s socioeconomic conditions and increases in population during the past few decades, the total number of patients with PD has correspondingly increased, which poses a significant challenge in a rapidly aging population (6). The increased proportion of elderly people, longer life expectancy, improved medical facilities, and growing industrialization in China have contributed to a further increase in PD prevalence, which is associated with high treatment and direct healthcare costs. To date, the estimated total number of patients with PD in China could be as high as 3.62 million. PD affects not only the quality of life (QOL) of aging populations but also their life spans, requiring a greater level of care and attention. From 1999 to 2019, the mortality from PD increased from 5.4 (95% confidence interval [CI]: 5.3–5.5) per 100,000 population in 1999 to 8.8 (95% CI: 8.7–8.9) per 100,000 population in 2019 in the United States (7). Currently, the pathogenesis, diagnosis, prognosis, and management of PD are the important aspects of the “one body, two wings” scheme of the China Brain Project.

Generally, PD is characterized by cardinal motor symptoms including tremor, bradykinesia, rigidity, and postural instability; a diverse range of non-motor disorders such as rapid eye movement sleep disorder, anosmia, constipation, depression, cognitive impairment, and dysautonomia; and dysfunction of the autonomic nervous system and enteric nervous system, including hyperhidrosis, dysuria, and orthostatic hypotension (8). PD is referred to as the complex disease in clinical practice (9) and its clinical features impact the QOL of PD patients. Currently, the clinical diagnosis of PD is based on aforementioned typical motor symptoms and novel diagnostic biomarkers such as imaging markers, fluid and tissue α-synuclein markers have been developed (5). However, multimorbidity of non-motor disorders acts synergistically to heighten the risk of adverse outcomes for patients with PD, which usually appear 20 years before the onset of motor symptoms (10). To date, an accurate diagnosis of PD remains challenging and the characterization of the earliest stages of the disease is ongoing.

Li et al. found that the five highest frequencies of non-motor complications in PD patients are sleep disorders, depression, lower urinary tract symptoms, Alzheimer’s disease, and constipation in the early stage of PD, making both diagnosis and treatment difficult (10). Pathologically, two major hallmarks of PD are the progressive loss of dopaminergic neurons in the substantia nigra pars compacta and the formation of Lewy bodies and Lewy neurites. The main toxic component of Lewy bodies is the protein α-synuclein which plays a pivotal role in PD pathogenesis. There are several key molecular pathogenic mechanisms—including genetic factors, α-synuclein aggregation, impairment of protein clearance, mitochondrial dysfunction, ferroptosis, neuroinflammation, and oxidative stress—involved in PD pathogenesis (11–13). The exact PD pathogenesis is yet to be completely elucidated owing to the multifactorial nature of the disease, leading to the lack of disease-modifying treatment strategies. Current pharmacological and non-pharmacological treatments such as dopamine replacement therapy with levodopa are mainly focused on relieving the symptoms but have multiple side effects (14). Although highly efficacious symptomatic treatments were successfully developed and adopted in clinical practice for PD, these therapies are ineffective in restoring dopaminergic neurons and stopping or slowing disease progression. Given that the 10-year prognosis for death or life-limiting disability for those who are diagnosed as PD is upwards of 80% (9), there is a desperate need for curative treatments that go beyond symptom management. The ability to stop, prevent, or mitigate progression remains an urgent unmet challenge in PD research at present. Many novel anti-Parkinson’s therapeutic agents such as anti-apoptotic agents, monoamine oxidase inhibitors, microRNAs, and viral vector gene therapy have shown general success in preclinical studies across different animal models (15, 16), but little benefit has been reported in human intervention studies or clinical trials. Thus, a better understanding of the PD pathogenesis underlying these symptoms and identification of new therapeutic targets are needed to develop effective therapeutic approaches for PD.

## Altered Microbiota Profiles and Functions in PD

The strong connection between the gastrointestinal environment status and central nervous system (CNS) function—the brain–gut axis—has received increasing attention in the scientific and medical communities. The normal bidirectional communications in the gut–brain axis play vital roles in maintaining homeostasis of the gastrointestinal tract and brain; disruption of this complex relationship has been shown to be associated with the pathogenesis of several neurological disorders including PD (17). Traditionally, the cross-talks along the gut–brain axis have been viewed primarily as mediated by neuro-hormonal factors or inflammatory mediators (18). Recent research has identified another key factor that could influence both gut and brain function: the gut microbiota (17, 19).

### Altered Gut Microbiota Characteristics in PD

The gut microbiota—which is also referred to as the second brain, forgotten organ, individual’s identity card, and host’s fingerprint—may affect brain activity through the microbiota–gut–brain axis under both physiological and pathological conditions (20, 21). With the development of omics techniques, the gut microbiota is considered as one of the important factors in regulating the gut–brain interactions. There is growing acceptance that the gut can influence CNS function and vice versa giving rise to the communication pathway—the microbiota–gut–brain axis. Recent breakthroughs have been made in understanding the intestinal origin of PD. Evidence indicates that gut dysbiosis might trigger and/or exacerbate the progression of PD (17, 22–24), which has opened up new avenues to explore the PD pathogenesis and its novel treatment approaches. Many studies have highlighted that the gut microbiota can regulate the gut–brain axis through endocrine, immune, and direct neural mechanisms, thus supporting the hypothesis that the pathological process of PD spreads from gut to brain (25, 26). This notion is supported by pathophysiologic evidence: the major component of Lewy bodies, α-synuclein, has been found to be accumulated abnormally in the enteric nervous system (ENS) (27). Holmqvist et al. showed that injection of human α-synuclein fibrils into the gut tissue of healthy rodents is sufficient to induce aggregated α-synuclein pathology within the vagus nerve and brainstem, which provided the first direct experimental evidence that α-synuclein can propagate from the gut to the brain (28). Recently, another study also demonstrated gut-to-brain spread of pathological α-synuclein fibrils following their injection into the mouse muscularis layer of the pylori and duodenum, but not when the mice were subjected to truncal vagotomy following α-synuclein fibril injection (29).

Cersosimo and Benarroch also found that gastrointestinal dysfunctions including constipation, bloating, nausea, dysphagia, sialorrhea, vomiting, and gastroparesis are common in PD (i.e., seen in over 80% PD cases) (30) and can begin decades before the onset of motor symptoms. Adams-Carr et al. demonstrated that people with constipation are at a higher risk of developing PD than those without and that constipation can predate diagnosis of PD by over a decade (31). These gastrointestinal symptoms are strongly associated with α-synuclein-related neurodegenerative changes in the ENS (32). The deposition of α-synuclein is not only found in the gastrointestinal tract of PD patients with gastrointestinal symptoms (33) but also in the gastrointestinal tract of patients before they present with PD-related motor symptoms (34). This indicated that α-synuclein pathology is likely initiated in the ENS first, and then spreads to the CNS via the vagus nerve (35). These findings strengthened Braak’s hypothesis in that PD pathology may originate in the periphery and gradually propagate to the brain, where it eventually leads to clinically confirmed PD. Therefore, the concept of “gut-originality” is now widely accepted in present PD pathogenesis research and recent accumulating evidence has shown that gut microbiota may play a key role in the progression of PD.

In the past decade, many case-control studies in different regions including Western countries and China have investigated the overall structure of gut microbiota in PD patients, and explored the correlations between gut microbiota and PD clinical characteristics. Elucidating the alterations of the gut microbiota will provide a foundation to improve our understanding of the PD pathogenesis and support the potentially microbiota-modifying therapeutics. Human α-synuclein overexpressing (ASO) mice that received fecal microbiota transplantation from PD patients showed increased severity of manifestations than those animals that received fecal microbiota from healthy individuals, which provided direct evidence that dysbiosis of the gut microbiota may play a causal role in the development of PD.

In fact, the alterations of the gut microbiota in PD patients have been repeatedly demonstrated. A recent study also found significant microbiota differences in the PD appendix (36). However, the structure and composition of the gut microbiota are affected by various exogenous factors such as age, sex, body mass index, race/ethnicity, geography, diets, drugs, and life styles, which must be taken into account when evaluating the varying results of different studies. These external factors contributed to variations in diversity and composition of the gut microbiota, making it difficult to compare the gut microbiome composition across different studies and achieve a unanimous conclusion. Nevertheless, those case-control comparative studies can still provide some clues to explore the roles of the gut microbiota in the development of PD.

Gut microbiota dysbiosis was observed in patients with prodromal and/or clinically established PD when compared with well-controlled subjects. Using culture-independent high-throughput sequencing techniques, the overall structure and composition of the PD-associated gut microbiota have been investigated, and characteristics of the altered microbiota profiles in PD patients have been identified. While certain findings were replicated across several studies, various contradictory findings were reported. Generally, bacterial diversity is the most validated metagenomic marker of gastrointestinal health and metabolic disorders. Many previous studies have observed decreased bacterial diversity in PD patients, but higher α-diversity in PD patients (23, 37). One study also showed that β-diversity (between samples) differed between PD patients and controls (38). The loss of bacterial diversity, mainly measured with α-diversity indices such as Shannon and Simpson, has been linked to the clinical features of PD. A recent study performed by Heinzel et al. found that constipation, possible rapid eye movement sleep behavior disorder (RBD), physical inactivity, smoking, urate levels, and subthreshold parkinsonism might be particularly linked to the prodromal microbiome in PD. Constipation, physical inactivity, and occupational solvent exposure showed associations with bacterial α-diversity, while sex, physical inactivity, possible RBD, constipation, and smoking were associated with β-diversity. Age and urate-lowering medication were associated with both α- and β-diversity (39). However, Plassais et al’s investigation showed that the gut microbiome α-diversity is not a biomarker of PD (40). Regardless of these inconsistent results, the changed overall structure of the gut microbiota and its associations with PD clinical features implies that microbial dysbiosis may be a contributor to the etiopathogenesis of PD, even in its early stage.

As far as the altered composition of PD-associated gut microbiota was concerned, no consensus has emerged from existing human studies of PD and gut microbiota regarding which bacterial taxa are most relevant to PD. One meta-analysis of 15 case-control studies observed that Prevotellaceae, Lachnospiraceae, and Faecalibacterium were decreased significantly in patients with PD compared to healthy controls, while Ruminococcaceae, Bifidobacteriaceae, Christensenellaceae, and Verrucomicrobiaceae were enriched in patients with PD (41). Another recent meta-analysis of 10 relevant studies found an abundance of Megasphaera and Akkermansia, and reduced Roseburia in PD patients (42). To date, the most consistently shown PD-related changes of gut microbial composition include an increase in the relative abundances of Verrucomicrobiaceae and Akkermansia and a decrease in Prevotellaceae and Prevotella (38).

Prevotella, a highly specific prodromal marker of PD, has been associated with RBD (43) and with progressive PD motor symptoms over 2 years (44). A previous study showed that constipation is lowest and subthreshold parkinsonism is least frequent in individuals with the Prevotella-enriched enterotype (39). In addition, constipation severity is significantly correlated with the decrease of Blautia and Faecalibacterium, short chain fatty acids (SCFAs)-producing taxa that can exert positive effects on the intestinal mucosa (45) and which are decreased in PD. In contrast to PD, SCFA-producing bacteria in one study were not decreased in idiopathic RBD, which suggested that a decrease of SCFA-producing bacteria may be a prerequisite for the development of PD (46). Another specialized mucin-degrading Verrucomicrobiaceae genus, Akkermansia, is a potential next-generation microbe that has anti-inflammatory properties and is responsible for good health (47, 48). Akkermansia is associated with enhancement of wound healing, augmented antitumor responses, protection against obesity, and induced intestinal adaptive immune responses during homeostasis (49–52). However, Akkermansia cannot always be considered a potentially beneficial bacterium, because several studies have consistently reported a greater abundance of Akkermansia in the fecal samples of PD and AD patients than that in healthy controls (53–56).

Amorim Neto et al. observed that A. muciniphila (typical strain) induced mitochondrial calcium overload and α-synuclein aggregation in an enteroendocrine cell line (57). The PD-associated gut dysbiosis, such as increased Akkermansia and decreased SCFAs-producing bacteria, can increase the intestinal permeability and intestinal inflammation, which subsequently facilitates exposure of the intestinal neural plexus to toxins such as lipopolysaccharide (LPS) and pesticides; this can lead to abnormal aggregation of α-synuclein fibrils and generation of Lewy bodies (22). Choi et al. demonstrated that oral administration of Proteus mirabilis, increased markedly in PD mouse models, was sufficient to provoke selective death of dopamine neurons and motor deficits in mice, accompanied by neuroinflammation and accumulation of aggregates of α-synuclein in both the colon and brain (58). One gastric pathogen, Helicobacter pylori, was found to be associated with increased severity of motor dysfunction, decreased dopamine levels in the brain, and decreased levodopa absorption as well as autoimmune and inflammatory reactions (59).

Although the above case-control studies identified several key functional bacteria in the PD-associated gut microbiota, these cross-sectional gut microbiome studies could still not establish a causal relationship between variations in the gut microbiome and PD. Moreover, little is still known about whether such alterations precede disease onset and how they relate to risk and prodromal markers of PD. This knowledge gap greatly restricts the application of those key intestinal functional bacteria in the non-invasive diagnosis and microbiome-targeted treatment for PD.

Recently, a longitudinal study found that microbiota differences (e.g., in Roseburia, Prevotella, and Bifidobacterium) detected at baseline can be replicated at a follow-up timepoint 2 years later, and that there might be changes in gut microbiota composition in patients with faster disease progression (44). In addition, these altered key functional bacteria or biomarkers are correlated with non-motor symptoms, disease duration, treatment options, and even cognitive impairment (23), which suggest that gut microbiota can participate actively in the process of PD. Prospective long-term longitudinal microbiome studies are needed to monitor disease progression and characterize alterations in the taxonomic composition of the microbiome that lead to, or might even define, the disease state. However, exactly how the gut microbiota may impact PD-related symptoms remains unclear.

### Altered Gut Microbiota Functions in PD

While the present taxonomic changes of the gut microbiota in PD only provide information regarding correlations but not causation, a recent study has found casual associations between several gut bacteria such as Lentisphaera, Eubacterium hallii, Anaerostipes, and Clostridium sensu stricto 1 and PD through a Mendelian randomization approach (60). However, a correlation is not necessarily equal to causation, as just define how one variable change relatively to another. In fact, disentangling cause and effect is difficult. Given that microbiota functions are conserved across taxonomic groups, they are much more informative than taxonomic data, since it is what the microorganisms do that we care about and not who they are.

Notably, microbial functioning analysis is more productive than a purely taxonomic approach to understanding the gut microbiome in PD. Recently, metatranscriptomics, metaproteomics, and metabolomics have been used to explore the functionality of the microbiota and, therefore, provides some strong insights into microbial activities in the gut and their associations with health and diseases. Different to metagenomics or 16S rRNA gene sequencing, the fecal metabolome provides a functional readout of microbial activity and can be used as an intermediate phenotype mediating host-microbiome interactions (61). Bacterial metabolites mirror the altered gut microbiota composition in patients with PD. Tan et al. found that fecal metabolites in PD were significantly different from that in healthy controls, with the largest effect size seen in an NMR-based metabolome (62).

Differentially abundant fecal metabolites including bioactive molecules with putative neuroprotective effects (e.g., SCFAs, ubiquinones, and salicylate) and other compounds (e.g., trimethylamine N-oxide, ceramides, and sphingosine) are increasingly implicated in neurodegeneration. SCFAs such as acetate, butyrate, and succinate are produced owing to the fermentation process of gut bacteria. Notably, accumulating evidence highlights the important functions of SCFAs in many neurological diseases such as multiple sclerosis, AD, and PD (46, 56, 63–65). Reduced levels of SCFAs were found in PD patients, which increased the incidence of endotoxin and neurotoxin, both potentially associated with the development of PD. In PD patients, low SCFAs are significantly associated with poor cognition and low BMI; lower butyrate levels correlate with worse postural instability-gait disorder scores (62). In addition, SCFAs can promote gastrointestinal motility and regulate the function of the enteric nervous system, which is why a reduction of SCFAs might contribute to the development of gastrointestinal motility disorders such as constipation in PD (64). Fecal SCFAs were found to be inversely correlated with several PD-related clinical variables such as the Non-Motor Symptoms Scale score, the Rome III constipation/defecation subscore, stool consistency associated with constipation on the Victoria Bowel Performance Scale, and the Geriatric Depression Scale-15 (66). Microbial SCFAs play both direct and indirect key roles in microbiota–gut–brain axis signal communication via regulation of the gut epithelial barrier and blood–brain barrier integrity, neuronal survival, inflammatory cascades, and endocrine signaling (67).

A reduction of SCFAs-producing bacteria leading to SCFAs deficiency may result in detrimental effects in PD patients, including gut leakiness, increased colonic inflammation, increased risk of α-synuclein deposition in the gastrointestinal tract, and microglial activation in the brain (68, 69). SCFAs were recently reported to have protective properties against dopamine and tyrosine hydroxylase depletion in the substantia nigra (70). Liu et al. also found that butyrate exerts protective effects against PD in mice via stimulation of glucagon like peptide-1 (71). This finding suggested that alterations to SCFAs may play a role in the pathophysiology of PD and increase of SCFAs can retard the development of PD.

Apart from altered SCFAs, microbial dysbiosis can also affect lipid metabolism, including an upregulation of bacteria responsible for secondary bile acid synthesis. Bile acids are produced in the liver from cholesterol and then metabolized by gut microbiota-derived enzymes into secondary bile acids (72). Li et al. found that microbially derived toxic bile acids such as deoxycholic acid and lithocholic acid are heightened in PD (36). The increases of deoxycholic acid and lithocholic acid can propel the accumulation of pathological α-synuclein aggregates, which can potentially propagate from the gut to the brain through retrograde transport (73). However, Castrocaldas et al. demonstrated that tauroursodeoxycholic acid can rescue mitochondrial function and prevent 1-methyl-4-phenyl-1, 2, 3, 6-tetrahydropyridine (MPTP)-induced dopaminergic cell death in different animal models of PD (74). Ursodeoxycholic acid is another secondary bile acid that has neuroprotective effects (75) and is currently being tested in clinical trials for PD (76), which can prevent the damaging effects of deoxycholic acid and lithocholic acid. In a chronic PD mouse model, pretreatment with tauroursodeoxycholic acid can protect against dopaminergic neuronal damage, prevent microglial and astroglial activation, as well as the dopamine and 3-4-dihydroxyphenulacetic acid reductions caused by MPTP. Pretreatment with tauroursodeoxycholic acid can prevent protein oxidation and autophagy, in addition to inhibiting α-synuclein aggregation (77). This finding suggests that fecal biliary abnormalities may also play a crucial role in PD pathogenesis, and targeting microbial-derived secondary bile acids may be a new avenue for the earlier PD diagnosis and alleviation of PD symptoms.

Other microbiota-derived metabolites, including proteolytic metabolism products (e.g. p-cresol sulfate) and tryptophan catabolites (e.g. kynurenine and indolelactic acid), have shown significant alterations in PD, which are also crucial metabolic events underlying PD. Other metabolites derived from gut microbiota-based metabolism of dietary polyphenols—e.g., 3,4-dihydroxybenzoic acid (3,4-diHBA), 3-hydroxyphenylacetic acid (3-HPPA), and 3-hydroxybenzoic acid (3-HBA) — have shown an in vitro ability to inhibit α-synuclein oligomerization and mediate aggregated α-synuclein-induced neurotoxicity (78). Chung et al. found that plasma levels of trimethylamine N-oxide (TMAO), a gut microbiota-derived metabolite, are associated with faster increases in levodopa-equivalent dose and tend to increase the risk for PD-dementia conversion, and can therefore be considered as a biomarker in early PD (79). Microbiota-derived metabolite studies can provide comprehensive biochemical underpinnings to unravel the underlying mechanisms of PD pathogenesis, offer biomarkers that reflect PD pathological processes, and possibly substantially improve therapeutic strategies against PD. Thus, alterations of the gut microbiota and its associated microbiota functions might modulate the microbiota–gut–brain axis, and play crucial roles in the pathogeneses of PD, which provide novel insights into PD pathogenesis and microbiome-targeted therapeutic options for PD.

## Mechanisms of Gut Microbiota in PD

**Figure 1:**
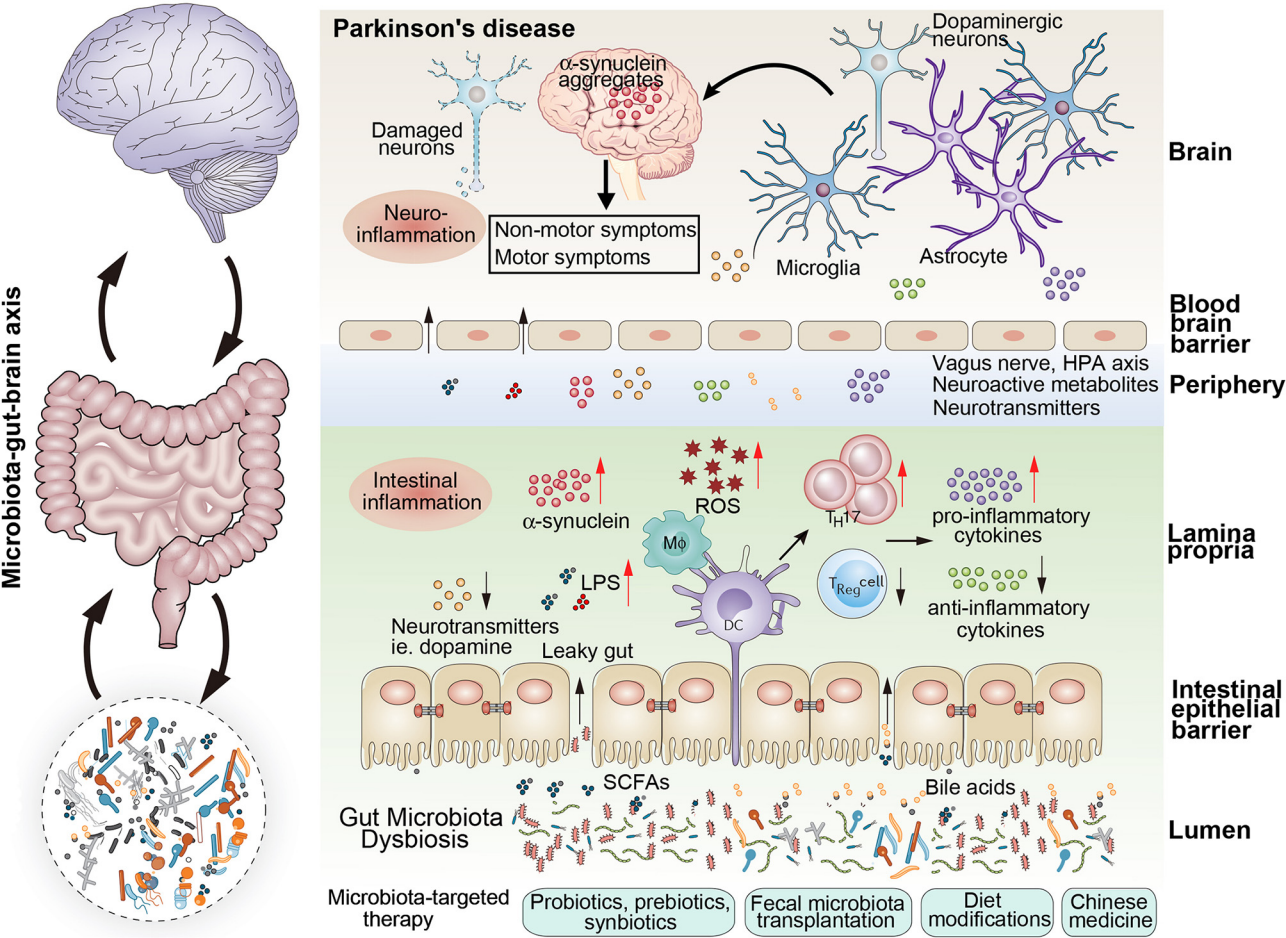
The schematic representation of the gut microbiota dysbiosis associated with parkinson’s disease. Microbiota dysbiosis plays vital roles in the occurrence and developemnt of parkinson’s disease. Dysbiotic gut microbiota was associated with the increased intestinal permeability, aggravated neuroinflammation, abnormal aggregation of α-synuclein fibrils, oxidative stress, and decreased neurotransmitter production, which played vital roles in the ocurrence and development of Parkinson’s disease. Several microbota-targeted therapies can be used to modify the composition of the gut microbiota to revert a dysbiotic condition: probiotics, psychobiotics, prebiotics, synbiotics, postbiotics, fecal microbiota transplantation, dietary modifications and Chinese medicines.

As mentioned above, dysbiotic gut microbiota are understood to play crucial roles in the occurrence and development of PD by increased intestinal permeability, aggravated neuroinflammation, abnormal aggregation of α-synuclein fibrils, oxidative stress, and decreased neurotransmitter production (**Figure 1**).

### Increased Intestinal Permeability

Increased intestinal permeability, also known as “leaky gut,” has been detected in patients with PD—even in cases with minimal or no gastrointestinal manifestations (80). Pathogenetically, the gastrointestinal tract is proposed to be the initial site of pathological changes in PD. During dysbiosis, the destruction of intestinal epithelial barrier integrity can influence the initial step of the following cascade of neurodegeneration in PD. Accumulating evidence has emphasized the important role of the intestinal barrier and intestinal permeability on health and disease. Forsyth et al. found that increased intestinal permeability in PD patients, which is a feature of intestinal barrier function, strongly correlated with markers of increased exposure to endotoxin, and with a marker indicating increased oxidative stress burden in the intestine, which together may be responsible for the abnormal accumulation of α-synuclein in enteric neurons (81). Fecal zonulin and α-1-antitrypsin, markers of intestinal barrier permeability, have been found to be increased in PD patients (82, 83). In addition, reduction of tight junction proteins (TJs) expression in PD patients has been linked with increased intestinal permeability. The expression of TJs such as occludin, but not of zona occludens 1 (ZO-1) decreased significantly in lysates of sigmoid/descending colon biopsies of PD patients (84). Perez-Pardo et al. reported a reduction of ZO-1 immunolabeling in sigmoid colon biopsies from PD (85). However, the elevated fecal markers were not associated with other clinical features of PD, which suggested that a PD subpopulation should be considered when enrolling PD cohorts.

Several animal studies have provided causal evidence that intestinal impairments can trigger PD-related pathology. Kelly et al. demonstrated that increased intestinal permeability correlated with the presence of α-synuclein aggregates in a mouse model of PD (86). Another previous study found that increased intestinal permeability triggered the aggregation of α-synuclein in enteric neurons and dopaminergic neurons of the substantia nigra, ultimately leading to neuronal degeneration in aged α-synuclein transgenic mice (87). Further, in ASO mice, LPS injection reduced intestinal barrier integrity and expedited the onset of motor symptoms (88). Bhattarai et al. found that chronic administration of rotenone results in disrupted colonic epithelial permeability and development of motor symptoms only in conventionally raised mice but not in germ-free mice. Those results implied that gut microbiota are not only required but may in fact be the mediators of the effect of environmental toxins on intestinal permeability and the development of motor symptoms (24). Polymannuronic acid can greatly increase the integrity of intestinal barrier and blood–brain barrier as indicated by increased expressions of TJs in both mice colon and substantia nigra pars compacta in PD mice model, which can improve motor functions by preventing dopaminergic neuronal loss (89). The important roles of intestinal permeability have been highlighted in PD initiation, which paves the way for novel PD therapy targeting the restoration of the intestinal barrier. Indeed, future studies are still needed to determine whether increased intestinal permeability plays a causative role in PD initiation or is a consequence of PD pathogenesis.

### Inflammation

Inflammation, the hallmark of PD, has been linked to the development of non-motor symptoms in PD, which greatly impact patients’ QOL and can often precede motor symptoms. Mounting evidence has documented increased levels of a variety of inflammatory molecules in the brain and cerebrospinal fluid as well as blood from PD patients such as IL-1β, IL-2, IL-6, IL-10, TNF-α, CCL5, and CRP (90, 91). Several studies have found that calprotectin, a fecal marker of intestinal inflammation, was significantly elevated in PD patients compared to age-matched controls (83, 92). The level of fecal calprotectin correlates well with macroscopic and histological inflammation as detected by colonoscopy and biopsies, respectively. Another previous study observed increased fecal calprotectin levels in patients with inflammatory bowel disease (IBD) (93), and epidemiological data indicate a link between IBD and PD (94, 95). The detection of the fecal calprotectin may be a useful tool to detect the signs of gut immune system activation present in PD patients, possibly in the early stage of the disease (92). Klingberg et al. found that increased gut dysbiosis was associated with elevated fecal calprotectin (96). In fact, intestinal inflammation in PD is related to gut dysbiosis and gut permeability such that it can contribute to disease pathogenesis. During gut dysbiosis, the metabolites or components of pathogenic bacteria may cause inflammation, while the metabolic changes in the symbiotic bacteria can regulate inflammation, which result in PD pathology together.

Recent investigations have suggested that gut dysbiosis is linked to aberrant immune responses, which not only increases intestinal inflammation but also increases microglial activation and neuroinflammation (97, 98), which are central events in the pathogenesis of PD (99). As mentioned above, intestinal barrier deficiency can increase systemic exposure to inflammatory microbial products such as LPS, inducing intestinal inflammation, microglial activation, neuroinflammation, and oxidative stress and in turn neuronal pathological α-synuclein aggregates. LPS is a potential influence on brain glia originating in the gut microbiota, while depletion of gut bacteria reduces microglia activation (100). Hasegawa et al. found that plasma level of the LPS-binding protein was decreased in PD patients (101), suggesting more LPS reaches the CNS. Evidence from animal models has shown that LPS may not only enhance the inflammatory response in the CNS of PD patients but also accelerate the neurodegenerative process by the effects on α-synuclein (102). α-synuclein‐induced microglial activation (neuroinflammation) would potentiate further α-synuclein aggregation and propagation specifically in the nigrostriatal dopaminergic neurodegeneration, thus contributing to PD progression (100). The abundance of LPS-producing Bacteroides was found to be correlated with the plasma levels of TNF-α in Taiwanese patients with PD (91). Lin et al. found that the relative abundances of Verrucomicrobia and Bacteroides correlated with elevated TNF-α and IFN-γ in patients with PD, suggesting the development of a systemic sub-inflammatory status associated with gut dysbiosis (91). Sampson et al. revealed that gut dysbiosis or specific gut pathogens can promote neurodegeneration and motor deficits by activating neuroinflammation in an ASO mouse model (100), that can be used as potential therapeutic targets for PD treatment. In addition, decreased SCFAs also downregulates regulatory T cells and upregulates Th17 cells, which fails to suppress neuronal inflammation and ultimately leads to neurodegeneration (22, 103). Singh et al. also found that increased inflammation and decreased innate lymphoid cells (ILCs) in DJ-1−/− mice, which can be regulated by intestinal bacteria such as Alistipes and Rikenella (104).

Microbiota-modifying therapies may provide benefits to PD patients, but studies are lacking. Strong epidemiological evidence has suggested that smokers and coffee drinkers have a reduced risk of PD, possibly by shifting the composition of gut microbiota toward improved barrier function and decreased release of pro-inflammatory cytokines from the gut into the bloodstream, which likely reduces neuroinflammation (105). In one study, IBD patients who received anti-TNF therapy had a 78% reduction in the incidence of PD, suggesting that suppressing peripheral inflammation can protect against PD. In a rotenone-induced PD mouse model, fecal microbiota transplantation (FMT) could suppress inflammation mediated by the LPS-TLR4 signaling pathway both in the gut and brain (106). Sun et al. also found that FMT can correct the gut microbiota dysbiosis, reduce the activation of microglia and astrocytes in the substantia nigra, suppress neuroinflammation, and reduce TLR4/TNF-α signaling in the gut and brain (107). Another study found that acupuncture can protect dopaminergic neurons and enhance motor function, which may be associated with the regulation of gut dysbiosis and thus the inhibition of neuroinflammation in PD mice (108). Hence, further studies aimed to modify gut dysbiosis to suppress intestinal inflammation and neuroinflammation can potentially attenuate the neurodegenerative process in PD.

### α-Synuclein Pathology

Inflammation and α-synuclein misfolding are both key pathological mechanisms underlying α-synucleinopathies such as PD (109). Aggregation of α-synuclein is central to the pathogenesis of PD. α-synuclein consists of 140 amino acids, and the gene encoding it—synuclein α (SNCA)—comprises five exons and is located on chromosome 4q21.3-q22 (110). α-synuclein exhibits characteristics of prion-like protein during PD pathogenesis, with the misfolded α-synuclein turning the endogenous physiological protein into a pathogenic protein (26). Alterations in α-synuclein dosage lead to familial PD, while α-synuclein also contributes to fibrilization of amyloid-β and tau, two key proteins in AD, which suggest a central role for α-synuclein toxicity in neurodegeneration (111). The misfolding of α-synuclein into aggregates within nerve cells may contribute to neurodegeneration. Besides α-synuclein aggregation in PD patients, a previous study also found that α-synuclein fibrils are frequently observed in the brain even in healthy elderly subjects without motor or cognitive impairment, and sometimes in the intestinal neural plexus (22). The presence of aggregated α-synuclein in sigmoid colon biopsies may have 100% sensitivity and specificity for patients later developing pathologically advanced PD (112). The drugs designed to treat or prevent PD are focused on the prevention or elimination of α-synuclein aggregation; however, no successful cases have yet been reported yet.

The interaction between α-synuclein aggregation and the gut microbiota in PD is receiving increasing attention. Recent evidence suggests that α-synuclein aggregation may begin in the gut and gradually travel to the brain along the vagus nerve, with altered gut microbiota being a potential trigger for α-synuclein misfolding (29, 113, 114). Gut dysbiosis cause misfolding and abnormal aggregation of α−synuclein in the intestine, which can be transported from the ENS to CNS. Several reports have observed that gut microbiota play an important role in regulating misfolded and abnormal aggregation of α-synuclein and the gut–brain axis (115). Generally, the gut dysbiosis in PD can affect the gastrointestinal mucosal barrier, leading to the translocation of bacteria or their products such as LPS. These bacterial products can increase oxidative stress and intestinal inflammation, which in turn increases the intestinal permeability, and increases the ability of α-synuclein to communicate with the ENS.

LPS, bacterial products from gram-negative bacteria that are increased in people with PD, can increase the nitration and oligomerization of α-synuclein by increasing the level of inducible nitric oxide synthase. In addition, LPS can downregulate TJs such as occludin in the intestinal epithelial cells and upregulate TNF-α, which activates macrophages and promotes the expression of α-synuclein, as observed in a mouse model (116). In rodent models, LPS can increase intestinal permeability and α-synuclein expression in the large intestine, which provides direct evidence between gut dysbiosis and α-synuclein pathology (86, 88). Other bacterial metabolites such as SCFAs, especially butyrate, serve an important role in maintaining intestinal integrity, and a lack of SCFAs can increase intestinal permeability. The decreased SCFA-producing bacteria in PD patients can lead to disruption of the intestinal mucosal barrier, and this serves as a prerequisite for entry of α-synuclein into the ENS to maintain excessive α-synuclein expression or even promote its misfolding (117). Therefore, the altered gut microbiota profiles and their bacterial metabolites can influence α-synuclein expression, misfolding, and transportation in the early stage of PD, which can be used as potential targets to antagonize α-synuclein pathology.

Various studies have targeted α-synuclein directly at different stages of its synthesis and action as a potential therapeutic intervention. In vitro bacterial treatments and in vivo FMT also support the role of the gut microbiome in α-synuclein aggregation, gastrointestinal inflammation, and motor symptom development (88, 107). A previous study observed that ASO mice that received healthy gut microbiota showed improved motor deficits by down-regulating α-synuclein expression. Zhong et al. found that FMT can alleviate physical impairment, decrease fecal SCFAs and the expression of α-synuclein, and inhibit the activation of microglia in a mouse model of PD (118). Clinical observations found that probiotics can reduce the production of α-synuclein aggregates by inhibiting the reactive oxygen species level and finally alleviating the condition of patients with PD (119). Recently, Wang et al. demonstrated that Lactobacillus plantarum DP189 can reduce α-synuclein accumulation in the substantia nigra of the PD mouse model via inhibiting NLRP3 inflammasome, which can resist the development of PD (120, 121). Furthermore, previous studies have reported that coffee contains possible neuroprotective compounds such as caffeine and neuroprotective polyphenols (122, 123). Coffee can ameliorate motor deficits and dopaminergic neuronal loss, and reduce α-synuclein aggregation in MPTP-induced mice via regulating gut microbiota (124). Coffee can increase the level of anti-inflammatory Bifidobacteria and decrease the levels of Clostridium spp. and Escherichia coli that invade the gut mucosa in PD (125). These observations suggested that reducing α-synuclein expression, aggregation, or propagation by targeting gut microbiota modulation may represent a potential novel therapeutic option for PD treatment.

### Oxidative Stress

Oxidative stress plays a vital role in the degeneration of dopaminergic neurons in PD. An imbalance between reactive oxygen species (reflecting oxidative stress status) and antioxidant activity promotes inflammatory conditions in PD, which can create a vicious cycle and worsen neuronal cell death. Oxidative stress is considered the common underlying mechanism for cellular insult and apoptosis of dopaminergic neurons, regardless of whether PD is genetic or idiopathic (126). Thus, therapies targeting the suppression of oxidative stress may delay or reduce the severity of PD (127). It is increasingly accepted that oxidative stress may be aggravated by concomitant PD-associated gut dysbiosis (128). Generally, healthy gut microbiota have an immense antioxidative and anti-inflammatory role, while gut dysbiosis can manifest low-grade inflammation, cellular degeneration, and an imbalance of cellular energy followed by an increasing oxidative stress state (129). An unbalanced cycle of oxidative stress caused by gut microbiota dysbiosis may have the effect of gradually promoting specific phenotype of PD.

Oxidative stress may activate enteric neurons and glial cells, contributing to accumulation and misfolding of α-synuclein in the ENS (130). An α-synuclein accumulation in the intestine can transfer to the brain, leading to microglia activation, causing an increase in oxidative stress that exacerbates neuroinflammation (68). As mentioned above, the increased intestinal permeability in PD can increase enteric and systemic exposure to LPS and other bacterial products, resulting in increased intestinal oxidative stress (131). Nishiwaki et al. reported that Akkermansia may increase intestinal permeability and expose the intestinal neural plexus to oxidative stress, which can lead to abnormal aggregation of prion-like α-synuclein fibrils in the intestine (46).

Recently, probiotics and prebiotics that can modulate the gut microbiota have been used to regulate oxidative stress in PD patients. Tsao et al. demonstrated that Lactobacillus salivarius AP-32 lowered oxidative stress and inflammation, increased serum antioxidant activity, altered the fecal microbiota composition, and increased the levels of SCFAs in fecal samples in a PD-like model (127). Wang et al. found that another strain of Lactobacillus, L. plantarum DP189, can delay the neurodegeneration caused by the accumulation of α-synuclein in the substantia nigra of PD mice via suppressing oxidative stress, repressing proinflammatory response, and modulating gut microbiota (120). Marsova et al. found that Lactobacillus can reduce the level of oxidative stress (reactive oxygen species) in the PD nematode model by regulating the Nrf2/ARE pathway (132). These probiotic supplements directly and indirectly suppress oxidative stress by upregulating antioxidant pathways and by increasing antioxidant capacity, which can influence PD pathology.

### Neurotransmitters

The gut microbiota produces many neurotransmitters found in the human brain such as dopamine, serotonin, gamma-aminobutyric acid, and norepinephrine (133–135) and have been shown to influence various neurodegenerative disorders including PD. These neurotransmitters are involved in brain functions such as emotion, movement, learning, and memory actively. Generally, the gut microbiota directly produces neurotransmitters by encoding genes for specific enzymes or regulates the host biosynthesis of neurotransmitters with bacterial metabolites (136). In addition, dietary tryptophan can be metabolized by gut microbiota, which can produce various metabolites such as kynurenic acid and quinolinic acid. Several studies found that kynurenic acid is neuroprotective owing to its anti-inflammatory properties in the intestinal lumen (137, 138), while quinolinic acid is a blood–brain barrier modulator (139). Previous studies suggested that a healthy gut microbiota is associated with balanced neurotransmitter levels in the host. Studies have demonstrated that the absence or deprivation of gut bacteria in both germ-free and antibiotic-treated mice can influence the concentrations of neurotransmitters and their precursors in the gut and blood, and alter the expression of neurotransmitter receptors within the brain (140, 141). An imbalance in neurotransmitters eventually affects the pathogenesis of neurological and psychological disorders.

Dopamine, a major neurotransmitter, plays a critical role in vital functions such as cognition, motivation, and voluntary motor movements both peripherally and centrally. Optimal dopamine bioavailability is essential for normal brain functioning and protection against the development of neurological diseases (142). Levodopa is widely used in the treatment of PD, which can cross the blood–brain barrier and can be transformed into dopamine in the brain, thereby alleviating the symptoms of PD (143). However, the bioavailability of levodopa, required to ensure sufficient amounts of dopamine will reach the brain (144). Growing evidence has indicated that the gut microbiota can influence drug pharmacokinetics directly or indirectly and correspondingly bioavailability, efficacy, or adverse effects (145, 146). The microbiota, and in particular microbiome-encoded enzymes, can alter the absorption, distribution, metabolism, and elimination of drugs to consequently enhance or dampen clinical response and adverse effects. van Kessel et al. found that bacterial tyrosine decarboxylases (TDC) can efficiently convert levodopa to dopamine. In situ levels of levodopa were compromised by the relative abundance of gut bacterial TDC in the patients with PD. They also found that the higher bacterial TDC at the site of levodopa absorption—the proximal small intestine—influenced the levels of levodopa in the plasma significantly (147).

The gut microbes belonging to the genera of Bacteroides, Prevotella, Bifidobacterium, Lactobacillus, Enterococcus, Clostridium, and Ruminococcus can regulate dopaminergic activity (142). van Kessel et al. also demonstrated that the TDC genes (tdc) can be found in more than 50 Enterococcus strains (mainly E. faecalis and E. faecium) and several Staphylococcus and Lactobacillus species, while lower plasma levels of levodopa in rats treated with levodopa/carbidopa correlated with the level of bacterial tdc in the jejunum (147). They also found important associations between gut bacterial tdc-gene abundance and anti-PD medication (147). In addition, reduced levodopa availability is found in H. pylori-positive PD patients, while eradication of H. pylori improves levodopa bioavailability and motor control. These findings may be explained by the observation that H. pylori can bind levodopa in vitro via surface adhesins (148). All these findings imply that the regulation of neurotransmitters with the specific bacteria from the gut microbiota will be a useful therapeutic strategy for PD treatment.

## Gut Microbiota as a Therapeutic Target for PD

As mentioned above, the gut microbiota is intimately connected to the occurrence, development and progression of PD, especially in the early stages. A better understanding of the microbiota–gut–brain axis in PD can provide an opportunity to monitor an individual’s health by manipulating the gut microbiota composition. Several approaches like administration of probiotics, psychobiotics, prebiotics, synbiotics, postbiotics, FMT, dietary modifications and Chinese medicines have been tried to mitigate the dysbiosis-induced ill effects and alleviate PD progression (**Figure 1**).

### Probiotics, Psychobiotics, Prebiotics, Synbiotics and Postbiotics

Probiotics are live microorganisms which when administered in sufficient amounts confer a health benefit on the host. Psychobiotics are a special class of probiotics that deliver mental health benefits to individuals (149). Psychobiotics are currently being investigated as direct and/or adjunctive therapies for psychiatric and neurodevelopmental disorders and possibly for neurodegenerative disease, and they may emerge as new therapeutic options in the clinical management of brain disorders. Prebiotics are mostly fibers that are non-digestible food ingredients that beneficially affect the host’s health by selectively stimulating the growth and/or activity of some genera such as Lactobacilli and Bifidobacteria (150). Synbiotics are the combination of prebiotics and probiotics, wherein the prebiotic component selectively favors a probiotic strain. Postbiotics are functional bioactive compounds, secreted by live bacteria or released after bacterial lysis, such as non-viable microbial cells, cell walls, lysates, fractions, secretions, components and metabolites (eg, SCFAs acetate, propionate, and butyrate) that endows healthiness to the host like live probiotic cells when received in adequate amount (151).

As mentioned earlier, lactic acid bacteria such as Bifidobacterium, Lactobacillus, and Streptococcus species, are by and large the commonly used probiotic strains in clinical practice. Previous study has found that the combination of L. acidophilus and B. infantis can alleviate the symptoms of bloating and abdominal pain in PD patients (152), while one probiotic mixture with four strains including B. bifidum, L. reuteri, L. acidophilus, and L. fermentum can decrease the movement disorders society-unified PD rating scale (MDS-UPDRS) scores (153). Lu et al. demonstrated that L. plantarum PS128 supplementation for 12 weeks with regular anti-parkinsonian medication improved the UPDRS motor score and QOL of PD patients (154). Other probiotic strains or mixtures can also help to relieve gastrointestinal dysfunctions such as constipation and defecation habits in PD patients (155–158). Of course, the precise mechanisms of probiotics against PD, although not yet clear, are most likely via multiple routes, i.e., oxidative stress, inflammatory and anti-inflammatory pathways, as well as apoptosis.

Several studies have provided important clues regarding the mechanism of action of probiotics in parkinsonism mouse models. Recently, L. plantarum DP189 was found to be an effective psychobiotic to reduce α-synuclein aggravation in MPTP-induced PD mice via regulating oxidative damage, inflammation, and gut microbiota dysfunction (120). One widely studied L. plantarum strain—PS128—can suppress glial cell hyperactivation, attenuate MPTP-induced oxidative stress and neuroinflammation, modulate the gut microbiota, promote intestinal motility and mucin production, and alleviate motor deficits and neurotoxicity in mouse models of PD (159–161). In germ-free mice, PS128 can increase the levels of both serotonin and dopamine in the striatum and improve anxiety-like behaviors (162). In a murine model of PD, selected lactic acid bacteria mixture including L. plantarum CRL 2130 (a riboflavin producer), S. thermophilus CRL 807 (an immunomodulatory strain), and S. thermophilus CRL 808 (a folate producer) improved motor behavior and neuroinflammation in PD (163). Another Lactobacillus strain, namely L. salivarius AP-32, could enhance the activity of host antioxidant enzymes via direct and indirect modes of action in a rat model of PD (127). Similarly, L.rhamnosus HA-114 treatment improved hippocampal-dependent cognition in a PD model (164).

Srivastav et al. found that a probiotic mixture containing LGG, B. animalis lactis, and L. acidophilus increases the butyrate level, and subsequently rescues the nigral dopaminergic neurons from MPTP and rotenone-induced neurotoxicity (165). Ishii et al. demonstrated that oral supplementation of probiotic B. breve strain A1 can improve facilitation of hippocampal memory extinction via restoration of aberrant higher induction of neuropsin in a PD mouse model (166). Beyond the commonly used probiotics, our previous study also found that probiotic Clostridium butyricum could improve motor deficits, dopaminergic neuron loss, synaptic dysfunction, and microglia activation in a PD mouse model via the gut microbiota–GLP-1 pathway (19). The probiotic metabolites such as butyrate, also referred to as postbiotics, exert protective effects against PD in mice via stimulation of colonic glucagon like peptide-1 secretion (71). Furthermore, Hsieh et al. showed that long-term administration of probiotics has neuroprotective effects on dopamine neurons and further attenuates the deterioration of motor dysfunctions in MitoPark PD mice (167). Indeed, the benefit of the probiotics or psychobiotics for PD treatment is eminent, which can be used as an adjunct therapeutic option for PD. However, the selected probiotic strains, adequate doses, and duration of administration influences the efficacy for PD treatment (168). Future studies, especially mechanistic explorations and randomized controlled trials (RCT), will likely provide adequate evidence for PD treatment, which will promote their future translational and clinical applications.

Prebiotics, mainly dietary fibers, have been used to mitigate various diseases such as gastrointestinal dysfunction and allergic disorders (169). To date, no studies have evaluated the effects of prebiotics in PD patients alone; however, the success of sodium oligomannate (GV-971) for mild-to-moderate AD, which targets the gut microbiota, has provided a promising avenue for mining prebiotics for PD (170, 171). The decreased SCFAs-producing bacteria in PD can be rectified by prebiotic fibers and in turn impact the regulation of inflammatory processes and intestinal barrier integrity. Of course, direct oral supplementation of butyrate leads to an increase in plasma concentration that may well result in direct actions in the brain (172). A study examining β-galacto-oligosaccharides (GOS), fructooligosaccharide (FOS), or placebo in a rat model reported alterations in pivotal receptors for synaptic plasticity and memory function (173). This finding suggested that prebiotics may play a role in the neurological preservation of the CNS. The combination of prebiotics with probiotics, i.e., synbiotics, may efficiently restore the eubiosis of gut microbiota and improve gastrointestinal functions, which can be beneficial for PD treatment.

Recently, Liu et al. found that polymannuronic acid plus L. rhamnosus GG as a novel synbiotic promoted their individual neuroprotection against PD, which could therefore be one of the ideal oral agents for PD therapy (174). An RCT found that the consumption of a fermented milk containing multiple probiotic strains and prebiotic fiber was superior to placebo in improving constipation in patients with PD (156). Recently, postbiotics have drawn attention because of their immunomodulatory, anti-inflammatory, anti-obesogenic, anti-proliferative, anti-hypertensive, hypocholesterolemic, and antioxidant activities (175), although the exact mechanisms have not been fully elucidated. Among these important postbiotics, SCFAs, were found to be related with the occurrence and development of PD. Previous studies have found that the administration of one dominant SCFA butyrate in PD animal models was reported to ameliorate motor impairment and dopamine deficiency, and inhibit neuroinflammation (71, 176, 177). These findings prove that butyrate might act as a potential therapy for PD patients (178). Thus, the roles of probiotics, psychobiotics, prebiotics, synbiotics and postbiotics reveal their potential therapeutic value for management of PD. Further studies, especially well-designed RCTs, are needed in more populations to determine the optimum formulae, efficacy, treatment modalities, treatment duration, and side effects of these agents.

### FMT

FMT has been regarded as a more comprehensive method to reconstruct the gut microbiota, by transplanting gut microbiota of healthy donors into patients’ intestines (117). This therapy can be traced back to 1700 years when it was used for the first time, when a Chinese medical scientist named Ge Hong treated patients with severe diarrhea or gastroenteritis (179). Gut dysbiosis mediates the progression of PD, suggesting that the restoration of gut microbiota may be an emerging effective therapeutic option for PD. The exploration of FMT as an effective therapeutic strategy in PD treatment has garnered considerable attention. At present, the application of FMT for PD treatment is still at the initial stage, and only a few case studies and small-sample studies have found some clinical efficacy of FMT. Kuai et al. found that FMT can alleviate the PD patient’s constipation symptoms and improve their motor and non-motor symptoms (117). One case study reported that FMT can significantly reduce the short-term constipation and tremor of lower limbs in patients with PD in China (180). Another case report showed that FMT via colonoscopy resulted in improvement of PD motor and non-motor symptoms at 6 months, including constipation (181). A large descriptive cohort study of 2010 patients who underwent FMT and received follow-up for more than 3 months found that FMT is a safe and effective method for the treatment of gastrointestinal dysfunction (182), and another study conducted by Li et al. reported similar results (183). Xue et al. showed that 15 PD patients who received FMT (10 patients via colonoscopy and five via nasointestinal route) reported improved sleep state, quality of life, anxiety and depression, and motor symptoms at 1 and 3 months of follow-up (184). However, the long-term curative effect of FMT for PD is still unstable, which might be associated with the donor’s fecal microbiota composition, delivery route, and persistence of microbiota reconstruction after FMT. Animal experiments also suggest that FMT is helpful for treating PD. The possible mechanisms of FMT are manipulation of gut microbial composition, gut barrier fortification, pathogen suppression, and immunomodulation. Sun et al. found that FMT from normal mice donors into the PD recipients can mitigate gut microbial dysbiosis, which can increase the levels of Firmicutes and Clostridiales and reduce Proteobacteria, Turicibacterales, and Enterobacteriales (107). In addition, FMT can alleviate physical dysfunction, boost the levels of striatal serotonin and dopamine, and inhibit neuroinflammation in PD mice. Sampson et al. found that colonization of ASO mice with microbiota from PD-affected patients enhances motor dysfunction and increase microglia activation compared to microbiota transplants from healthy human donors (100). A recent study by Zhao et al. showed that FMT treatment can correct the gut microbiota dysbiosis and improve symptoms in a rotenone-induced PD mouse model, wherein suppression of inflammation mediated by the LPS-TLR4 signaling pathway both in the gut and the brain possibly plays a significant role (106). Thus, FMT has great potential as a therapeutic modality for PD in the future. However, certain adverse events of FMT such as infection and sepsis, transmission of enteric pathogens, bleeding, cytomegalovirus reactivation, and pneumonia should not be ignored (185). There is a need for high-quality clinical trials with larger sample sizes to gather enough clinical evidence so that FMT can qualify for wider clinical application in PD.

### Diet Modifications

A growing body of epidemiological studies have reported that diet affects (positively or negatively) the onset of neurodegenerative disorders including PD. The amount, type, and balance of dietary macronutrients (carbohydrates, proteins, and fats); high consumption of vegetables, fruits, and omega-3 fatty acids; and healthy diet patterns such as the Mediterranean diet may have a great neuroprotective influence on PD (186). One systemic review of 64 studies found that the Mediterranean diet, high in fiber and polyphenols, is related to a lower risk of PD onset (187), while the Western diet, low in fiber, may correlate with an increased incidence of PD and exacerbates the severity of PD (188). O’Keefe et al. also demonstrated that westernized diet is associated with the morbidity and mortality of westernized diseases (189). Barichella et al. found that higher caloric intake of macronutrients and micronutrients correlate with worse PD-related symptoms (190); whereas, calorie restriction or dietary restriction activate key pathways that might be important for preventing or slowing down the progression of PD (191). Zapała et al. revealed significantly higher intake of margarine and red meat in the patients with PD relative to healthy controls (192). In contrast, the specific components of the Mediterranean diet such as fresh fruits and vegetables, nuts and other dried fruits, olive oil, wine, and spices are the reason of this positive effect (187). Consumption of flavonoid-rich foods and Polyunsaturated fatty acids can reduce the risk of developing PD. Thus, the types of diet may be one of the more critical triggers or therapy for PD.

Diet can directly or indirectly impact health via various mechanisms. Recent mounting evidence suggests that the effect of diet on brain health is not because of a diet-induced inflammatory response, rather because of the effect of the composition of the diet on the gut microbiome (193). Previous studies have found that diet may be the single greatest factor determining the structure and metabolic function of the gut microbiota (189, 194). Wu et al. demonstrated that long-term dietary patterns are linked to gut microbial enterotypes (194). An international review has provided irrefutable evidence from around the world that the human microbiome can be modified by dietary changes in children and adults (195). Generally, a healthy diet such as a Mediterranean diet can increase beneficial gut bacteria, which can correct the gut dysbiosis in PD and alleviate parkinsonism symptoms. High dietary fiber or carbohydrate polymers in the Mediterranean diet can be utilized by the gut microbiota to release metabolites such as SCFAs that have a beneficial effect on PD as they increase the motility of the gastrointestinal tract by modulating ENS activity.

Ketogenic diet, characterized by low carbohydrate and high fat with an adequate protein, is receiving acceptance as a potential therapy for PD (196). Shaafi et al. observed a beneficial influence of ketogenic diet on the motor function in a PD rat model (197). Ketogenic diet causes an increase in SCFAs and a decrease in γ-glutamyl amino acid by altering specific microbial diversity. Ang et al. found that ketogenic diets can alter the gut microbiota resulting in decreased intestinal Th17 cells, which contribute to a reduction in gastrointestinal inflammation (196, 198). However, no study has yet explored the effects of ketogenic diet on gut microbiome in PD patients (199).Moreover, high dietary intake of ω-3 polyunsaturated fatty acids can influence the gut microbiota composition, which have anti-inflammatory properties that can reduce oxidative stress and therefore reduce α-synuclein accumulation (200). An animal study also demonstrated that supplementation of ω-3 fatty acids is associated with restoration of disturbed gut microbiota caused by early-life stress (201). The ω-3 fatty acids deficient-diet lead to increased fear-induced freezing behavior, decreased sociability, and increased depressive behavior in the offspring when they became adults. In addition, coffee and caffeine in the diet have also been consistently correlated with decreased risk of PD, which can counteract shifts in the Firmicutes/Bacteroides ratio, resulting from a Western diet (202). Other studies have mentioned that the beneficial effects of caffeine in reducing PD risk may be associated with the alterations of the gut microbiota (105, 203).

Calorie restriction and fasting-mimicking diet show some neuroprotective effects against PD via increasing β-hydroxybutyrate, fibroblast growth factor 21, and ghrelin levels, which may be a result of changes in the composition of the gut microbiome (204, 205). Cox et al. found that calorie restriction slows age-related microbiota changes (reducing the level of Bacteroides) in an AD model in female mice (206) and confers improved long-term rehabilitation of ischemic stroke via gut microbiota (207). Calorie restriction selectively enriches Lactobacillus-predominant microbial communities and suppresses the expression of core microbial genes related to LPS biosynthesis, in addition to reducing LPS levels and systemic inflammation. In one recent study, Zhou et al. showed that FMT from normal mice with fasting-mimicking diet treatment into antibiotic-pretreated PD mice recovered the latter’s motor function, alleviated the loss of dopaminergic neurons in the substantia nigra pars compacta, and normalized dopamine and 5-hydroxytryptamine levels in the mice striatum, suggesting that its neuroprotective effects may be mediated by reshaping the gut microbiota to modulate microbial dysbiosis. The most striking findings from that study are that a fasting-mimicking diet can be a novel means of preventing and treating PD by promoting a favorable gut microbiota composition and metabolites, which suggests that gut microbiota is the mediator for the diet-microbiota–gut–brain axis (208). However, whether diet modifications can delay the progression of PD from the prodromal phase to the overt motor phase, and whether diet modifications can modify disease course, disease progression, and response to levodopa treatment in those who suffer from motor symptoms still need further exploration.

Although diet modifications or nutritional intervention cannot directly prevent or delay the progression of PD, it can influence both the microbiota–gut–brain axis by modifying the microbiota composition and the neuronal functions of the ENS and CNS to ameliorate the progression of PD pathogenesis (209). Clarifying these roles of dietary patterns in PD will be meaningful for future personalized-dietary interventions such as microbiota-directed diets in patients with PD.

### Chinese Medicines

During the past few decades, Chinese medicine has gained wider and increasing acceptance among both the public and medical professionals owing to its therapeutic efficacy for many conditions including PD. With the advantages of low cost, high safety, and high biological activity, Chinese medicine has great advantages in the prevention and treatment of PD. Traditional Chinese medicines, mainly Chinese herbs, have shown potential clinical efficacy in attenuating the progression of PD. Clinical studies have shown that Chinese herb formulas as adjuncts improved both motor and non-motor symptoms simultaneously, and reduced the dose of dopaminergic drugs and occurrence of dyskinesia, which suggested the neuroprotective roles of these herbs for PD (210). Generally, the neuroprotective effects of Chinese herbs are highly depended on mixed formulas, not on a single active constituent. To date, several constituents of Chinese herbs including resveratrol, curcumin, and ginsenoside have reported neuroprotective function (211). Growing evidence suggests that Chinese herbs and herbal extracts may help the recovery of dopamine neurons and have a positive effect on ameliorating PD in animal models (212).

Chinese medicine can combat PD through multiple pathways such as anti-inflammatory anti-oxidant pathways, alleviate mitochondrial dysfunction, regulate autophagy, inhibit endoplasmic reticulum stress, and modulate gut microbiota. Among these pharmacological mechanisms, regulating the gut microbiota has emerged as a new avenue to understanding traditional Chinese medicines for PD treatment. The interactions between gut microbiota and Chinese medicines can influence the therapeutic outcomes for PD, as gut microbiota can metabolize Chinese medicines to produce new absorbable active small molecules which have active pharmacological effects, while Chinese medicines can regulate the composition of gut microbiota and its metabolites (213, 214). Although there are no large-scale cohort studies and mechanistic research on Chinese medicine, gut microbiota, and PD, Chinese medicine with multiple benefits endowed by the gut microbiota has made it a potential therapeutic approach for prevention and treatment of PD and other neurodegenerative diseases.

## Perspectives

The gut microbiota is one of the important factors involved in maintaining host health and disease. Mounting evidence has suggested that gut dysbiosis is associated with the development and progression of PD. The acceptance of gut-origin hypothesis has highlighted the importance of gut microbiota in the prodromal stage of PD, which is often 10–20 years before the onset of motor symptoms. The dysbiotic gut microbiota (including altered microbial metabolites) may play crucial roles in the occurrence of PD via various mechanisms such as increased intestinal permeability, aggravated intestinal inflammation and neuroinflammation, abnormal aggregation of α-synuclein fibrils, imbalanced oxidative stress, and decreased neurotransmitters production. Although the cause-effect links between gut microbiota and PD remain unclear, emerging evidence from PD animal model studies support that the dysbiotic gut microbiota can aggravate PD pathology, while re-establishment of the gut microbiota can delay or correct the onset of PD. This suggested that the gut microbiota can be considered as a diagnostic tool and therapeutic target for PD. Thus, novel therapeutic options aimed at modifying the gut microbiota composition and modulation of microbiota–gut–brain axis using probiotics, psychobiotics, prebiotics, synbiotics, postbiotics, FMT, dietary modifications, and Chinese medicines can influence the initial step in the cascade of neurodegeneration in PD, representing a forward-looking approach for PD. However, there is still a long way to go before a cure for PD can be discovered. Future studies are essential to further elucidate the cause-effect relationship between gut microbiota and PD, improved PD therapeutic and diagnostic options, disease progression tracking, and patient stratification capabilities to deliver personalized treatment and optimize clinical trial designs.

## Author Contributions

ZL, MZ, YY, XL, XY, YC, LZ, and FC discussed the contents, wrote, reviewed, and edited the manuscript. The final manuscript was read and approved by all authors.

## Funding

This present work was funded by the grants of Key R&D Program of Zhejiang (2022C03060), Lishui & ZJU Cooperation Project (2018zdhz07), the S&T Major Project of Lishui (2017YSKZ-01, 2017ZDYF04 and 2017ZDYF15), the National Natural Science Foundation of China (81771724, 31700800, 81790631), Zhejiang Basic Public Welfare Research Project (LGF20H090016), the Research Project of Jinan Microecological Biomedicine Shandong Laboratory (JNL-2022033C), the Taishan Scholar Foundation of Shandong Province (tsqn202103119), the Nutrition and Care of Maternal & Child Research Fund Project of Guangzhou Biostime Institute of Nutrition & Care (2019BINCMCF045), the National S&T Major Project of China (2018YFC2000500), and the Foundation of China’s State Key Laboratory for Diagnosis and Treatment of Infectious Diseases.

## Conflict of Interest

The authors declare that the research was conducted in the absence of any commercial or financial relationships that could be construed as a potential conflict of interest.

## Publisher’s Note

All claims expressed in this article are solely those of the authors and do not necessarily represent those of their affiliated organizations, or those of the publisher, the editors and the reviewers. Any product that may be evaluated in this article, or claim that may be made by its manufacturer, is not guaranteed or endorsed by the publisher.
